# Pluripotency Gene Expression and Growth Control in Cultures of Peripheral Blood Monocytes during Their Conversion into Programmable Cells of Monocytic Origin (PCMO): Evidence for a Regulatory Role of Autocrine Activin and TGF-β

**DOI:** 10.1371/journal.pone.0118097

**Published:** 2015-02-23

**Authors:** Hendrik Ungefroren, Ayman Hyder, Hebke Hinz, Stephanie Groth, Hans Lange, Karim M. Fawzy El-Sayed, Sabrina Ehnert, Andreas K. Nüssler, Fred Fändrich, Frank Gieseler

**Affiliations:** 1 Clinic for Applied Cellular Medicine, UKSH, Kiel, Germany; 2 First Department of Medicine, UKSH, Lübeck, Germany; 3 Clinic for Conservative Dentistry and Periodontology, School of Dental Medicine, Kiel, Germany; 4 Siegfried Weller Institute for Trauma Research, BG Trauma Center, Eberhard Karls University Tübingen, Tübingen, Germany; University of Quebec at Trois-Rivieres, CANADA

## Abstract

Previous studies have shown that peripheral blood monocytes can be converted *in vitro* to a stem cell-like cell termed PCMO as evidenced by the re-expression of pluripotency-associated genes, transient proliferation, and the ability to adopt the phenotype of hepatocytes and insulin-producing cells upon tissue-specific differentiation. However, the regulatory interactions between cultured cells governing pluripotency and mitotic activity have remained elusive. Here we asked whether activin(s) and TGF-β(s), are involved in PCMO generation. *De novo* proliferation of PCMO was higher under adherent vs. suspended culture conditions as revealed by the appearance of a subset of Ki67-positive monocytes and correlated with down-regulation of p21^WAF1^ beyond day 2 of culture. Realtime-PCR analysis showed that PCMO express ActRIIA, ALK4, TβRII, ALK5 as well as TGF-β1 and the β_A_ subunit of activin. Interestingly, expression of ActRIIA and ALK4, and activin A levels in the culture supernatants increased until day 4 of culture, while levels of total and active TGF-β1 strongly declined. PCMO responded to both growth factors in an autocrine fashion with intracellular signaling as evidenced by a rise in the levels of phospho-Smad2 and a drop in those of phospho-Smad3. Stimulation of PCMO with recombinant activins (A, B, AB) and TGF-β1 induced phosphorylation of Smad2 but not Smad3. Inhibition of autocrine activin signaling by either SB431542 or follistatin reduced both Smad2 activation and Oct4A/Nanog upregulation. Inhibition of autocrine TGF-β signaling by either SB431542 or anti-TGF-β antibody reduced Smad3 activation and strongly increased the number of Ki67-positive cells. Furthermore, anti-TGF-β antibody moderately enhanced Oct4A/Nanog expression. Our data show that during PCMO generation pluripotency marker expression is controlled positively by activin/Smad2 and negatively by TGF-β/Smad3 signaling, while relief from growth inhibition is primarily the result of reduced TGF-β/Smad3, and to a lesser extent, activin/Smad2 signaling.

## Introduction

The use of adult stem cells has been a reasonable therapeutic option for many diseases. One such cell type with inherent stem cell-like features is the human peripheral blood monocyte [1, 2]. By initially inducing a process of *de*differentiation, which involved the macrophage-colony stimulating factor (M-CSF)/interleukin-3 (IL-3)-dependent generation of a subset of cells with transient *de novo* mitotic activity [3] and the re-activation of pluripotency-associated genes [4], we have generated from these cells a derivative termed “programmable cell of monocytic origin” (PCMO). These cells have been suspected to be less mature and hence more stem cell-like than other monocytes [4]. PCMO are prone to acquire functional activities of hepatocyte-like cells (NeoHeps) and insulin-producing cells upon stimulation with appropriate differentiation media *in vitro* and *in vivo* following transplantation into mice [3, 5].

PCMO appear to be reprogrammed differentiated cells as they re-express a series of stem cell markers including the pluripotency-associated genes Oct4 (particularly the pluripotency-associated A isoform) and Nanog [4]. Expression of both genes steadily increased during culture and peaked at days 4–5 [4]. Interestingly, this coincided with peak proliferative activity of a monocyte subset as measured by cell counting, thymidine incorporation [3], activation of the proliferation-associated extracellular signal-regulated kinase 1/2 (ERK1/2) [6], and the kinetics of cyclin D1 expression (A.H., unpublished observation). This may suggest the possibility that both responses, Oct4 and Nanog expression, and mitotic activity, are controlled by the same factors. With respect to biotechnology applications, a deeper understanding of the molecular mechanism(s) and factors governing plasticity and proliferative activity in PCMO could lead to strategies that allow for an increase in stem cell-likeness and PCMO numbers and hence quality and yield of the differentiated end product. In the past, two attempts were successful to increase proliferation of PCMO: i) The use of autologous serum rather than fetal calf or human AB serum [7] and ii) the addition of factors with growth-stimulatory activity such as exogenous EGF or HB-EGF [6]. Other possibilities to achieve this goal are the modification of substrate attachment or attachment-free (suspension) culture, and the removal from or neutralization in the conditioned medium of autocrine factor(s) with growth-inhibitory properties.

Stemness and self-renewal of human embryonic stem (ES) cells and the expression of pluripotency-associated genes such as Nanog is sustained by members of the TGF-β/activin/BMP family of growth and differentiation factors [8–12]. The activins (A, B, AB) and the TGF-βs (1, 2, 3) all signal through specific type II (ActRIIA, ActRIIB, and TβRII, respectively) and type I (ALK4 and ALK5, respectively) receptors and the canonical Smad pathway, comprising the receptor-regulated Smads, Smad2 and Smad3, and the common-mediator Smad Smad4 [12]. Intriguingly, activin signaling is required to maintain self-renewal and pluripotency of human ES cells and mouse EpiSCs by controlling Nanog and Oct4 expression [9, 10, 13], which results in a block of neuroectoderm differentiation of pluripotency cells [11]. Nodal/activin signal through ALK4 and Smad2, the primary downstream transcriptional factor of the nodal/activin pathway, is essential for maintenance of the human and mouse primed pluripotent stem cell state [14, 15]. C-terminal phosphorylation of Smad2 (Smad2C) and nuclear localization induced by activin, or nodal signaling were observed in undifferentiated human ES cells (where Smad2 binds directly to the Nanog promoter [16]) and decreased upon early differentiation [11]. Stem cells interpret and carry out differential nodal/activin signaling instructions via a corresponding gradient of Smad2 phosphorylation that selectively titrates self-renewal against alternative differentiation programs by direct regulation of distinct target gene subsets and Oct4 expression [17].

TGF-β1 is produced by every leukocyte lineage, including lymphocytes, monocytes/macrophages, and dendritic cells, and its expression serves in both autocrine and paracrine modes to control the differentiation, proliferation, and state of activation of these immune cells [18]. As outlined above, PCMO generation appears to resemble the process of reprogramming of somatic cells. In contrast to activins, TGF-β signaling appears to antagonize reprogramming efficiency during generation of iPS cells from fully differentiated somatic cells [19, 20]. With respect to growth, TGF-β is known to arrest monocytes/macrophages in G1 of the cell cycle [21–23], an effect which is mediated primarily through p21^WAF1^ [21–23] and Smad3 [24].

Given the stem cell-like features of PCMO, it is conceivable that similar regulatory loops as in ES cells might operate in PCMO. Since PCMO were negative for Nodal, Gdf3, Lefty A, and Lefty B (members of the TGF-β superfamily of ligands which signal through ALK4 or ALK5) [4], we hypothesised that endogenous activin and TGF-β signaling were responsible for regulation of pluripotency and growth, respectively, in standard PCMO cultures. This was given support by the fact that Nanog and Oct4 are activin target genes and by of our earlier observation that TGF-β1 levels in culture supernatants correlated negatively with PCMO growth [7].

In this study, we characterize in PCMO cultures the endogenous activin and TGF-β production as well as intracellular Smad2C/Smad3C formation as a marker for endogenous activity of the Smad signaling pathway and indicator of autocrine activin/TGF-β signaling during the monocyte → PCMO conversion. Using highly specific inhibition strategies, we go on to analyze the relative contribution of activin and TGF-β signaling for two cellular responses crucially associated with the PCMO stem cell-like phenotype, expression of pluripotency genes and mitotic activity.

## Materials and Methods

### Reagents

Recombinant human TGF-β1, activin A, activin B, activin AB, follistatin, and neutralizing monoclonal anti-TGF-β1,-β2,-β3 antibody (#MAB1835) were all from R&D Systems (Wiesbaden, Germany). The ALK4/5/7 inhibitor SB431542 was purchased from Calbiochem/Merck (Darmstadt, Germany) and dissolved in dimethylsulfoxide.

### Ethics Statement

For the generation of PCMO and PCMO-derived hepatocyte-like cells, human peripheral blood mononuclear cells were retrieved from buffy coats of healthy blood donors. The study has been approved by the institutional ethics committee of the Medical Faculty of the University of Kiel, Germany, Project AZ:A133/04 on February 17th, 2005 and informed written consent was obtained from all donors.

### Generation of PCMO

Human peripheral blood monocytes were retrieved from buffy coats of healthy blood donors and cultured on tissue culture plastic as described in detail earlier [3–7]. Briefly, mononuclear cells were isolated by density gradient centrifugation (Ficoll-Paque; Amersham Pharmacia Biotech AB, Uppsala, Sweden) and cultured in 6-well plates (Cell+, Sarstedt, Numbrecht, Germany) for up to 4 days in RPMI 1640 medium (Life Technologies, Karlsruhe, Germany), supplemented with 5 ng/ml of M-CSF and 0.4 ng/ml of IL-3 (both from R&D Systems), 90 μM 2-mercaptoethanol, and 10% human AB serum (Lonza, Verbier, Belgium). On the day of isolation (day 0), 1–2 h after plating, cultures were gently washed to enrich for adherent cells and fresh medium was added to the adherent cell layer resulting in enrichment of 70%–80% as tested by flow cytometry analysis of CD45+ and CD14+ cells. The following day (day 1), the medium containing residual non-adherent cells (mainly lymphocytes) was removed and replaced by fresh medium. A second change of medium occurred on day 3. Due to the initial presence in the cultures of lymphocytes, some assays were started only after their complete removal on day 1 to avoid contamination of the samples with non-monocyte-derived proteins/RNAs. For suspension culture, monocytes were cultured in wells, the bottom of which were covered with a thin layer of agarose as described previously [25]. In all experiments, efficient generation of PCMO was validated by cell morphology and the capacity to differentiate into hepatocyte-like cells (S1 Fig.).

### Immunofluorescence

The staining procedure for PCMO has been published earlier [6]. Briefly, cytospins of PCMO were fixed in 1% paraformaldehyde, blocked and incubated with anti-human CD14 antibody (BD Biosciences, Heidelberg, Germany) at room temperature for 2 h and Alexafluor 488–labeled secondary antibody (Life Technologies) for 1 h. Cells were permeabilized with Triton X-100 (0.5%), incubated overnight with the anti-human Ki67 (BD Pharmingen) followed by Alexafluor 555-labeled secondary antibody (Life Technologies). In all experiments, Ki67-positive cells were counted double-blind by two investigators in at least 4 visual fields per slide and related to the total cell count of CD14-positive monocytes in the same field.

### RNA isolation and quantitative RT-PCR

Total RNA isolation from PCMO and human peripheral blood monocytes was performed using the GeneJet purification kit (Fermentas, St. Leon-Rot, Germany). To remove genomic DNA, all RNA samples were treated with DNase I, and primers spanning multiple exon-intron boundaries were used. For reverse transcription, 1 μg of the total RNA was reverse transcribed to first strand complementary DNA using the High-Capacity reverse transcription kit (Applied Biosystems, Darmstadt, Germany). Gene expression was quantified by quantitative real-time RT-PCR (qPCR) on an iCycler (Bio-Rad, Munich, Germany) and iCycler iQ Real-Time Detection System software (Bio-Rad). Thermal cycling was 10 min at 95°C for enzyme activation, denaturation for 15 s at 95°C, 60 s annealing at 60°C, and 60 s extension at 72°C. A dissociation curve was performed for each product to assure the absence of primer dimers or nonspecific products. The following primers were used (5’-3’): ALK4: sense: accagctgcctccaggccaac, antisense: gtgctcaggctccttgaggtgac; ALK5: sense: gcgacggcgttacagtgtttc, antisense: atggtgaatgacagtgcggtt ALK7: sense: caacaacataacactgcaccttcc, antisense: tttcatgtcgcagcatgaccgtc; ActRIIA: sense: tttgcctggaatgaagcatg, antisense: agaagccagttcccatagg; TβRII: sense: agcagaagctgagttcaacct, antisense: ggagccatgtatcttgcagtt; activin β_A_: sense: ggagaacgggtatgtggaga, antisense: ggatggtgactttggtcctg; TGF-β1: sense: accatgccgccctccggg, antisense: tcagctgcacttgcaggagc. The primer sequences for Oct4A and Nanog were given elsewhere [4]. Relative quantification was performed by the ΔΔCt method. Expression data for the genes of interest were normalized with those for the housekeeping gene GAPDH.

### Western blotting, ELISA, and TGF-β bioassay

Following various lengths of culture, PCMO were washed with phosphate-buffered saline to remove non-adherent cells and lysed in PhosphoSafe lysis buffer (Merck). Crude cell lysates, or nuclear proteins isolated with a commercially available kit (Thermo Scientific, Rockford, IL) were separated by sodium dodecylsulfate polyacrylamide gel electrophoresis, transferred to PVDF membranes (Immobilon P), and were probed with primary antibodies. Immunoreactive bands were detected by enhanced chemiluminescence. Primary antibodies used were p21^WAF1^ (#610233, BD Biosciences), Smad2 (Zymed Lab. Inc., Berlin, Germany), phospho-Smad2 (Ser465/Ser467) (#3101, Cell Signaling Technology, Heidelberg, Germany), Smad3 (Santa Cruz Biotechnology), phospho-Smad3 (Ser423/Ser425) (#9514, Cell Signaling Technology), Oct4 (# sc-5279, Santa Cruz Biotechnology), Nanog (Abcam, Cambridge, UK), α-tubulin and β-actin (Sigma, Deisenhofen, Germany). Secondary antibodies were obtained from GE Healthcare (Buckinghamshire, UK). Some blots were subjected to densitometric analysis using NIH imageJ. For better quantification of p-Smad2C, p-Smad3C, and p21^WAF1^ levels in PCMO, we employed in some experiments the following PathScan Sandwich ELISA Kits from Cell Signaling Technology: Phospho-Smad2(Ser465/467) #7348, Phospho-Smad3(Ser423/425) #12003, Total p21 Waf1/Cip1 #7167, and Total α-Tubulin #7944.

Activin A was measured with the Human/Mouse/Rat Activin A Quantikine ELISA Kit (R&D Systems, #DAC00B). The total amount of TGF-β (active + latent) was measured with the human TGF-beta 1 DuoSet ELISA (R&D Systems, DY240) following acid activation of the culture supernatants. The levels of bioactive TGF-β were measured with TGF-β reporter cells (MFB-F11, kindly provided by Dr. I. Tesseur) as described elsewhere [26]. Briefly, MFB-F11 cells were plated in 96-well plates (50,000 cells/well) and incubated for 24 h with 100 μl Medium (DMEM supplemented with 100 U/ml penicillin, 100 μg/ml streptomycin, 15 μg/ml hygromycin B). For measuring the levels of bioactive TGF-β, cells were stimulated for 48 h with 50 μl of the culture supernatant to be analyzed. Acid activated aliquots of the same supernatants were measured in parallel to obtain the amounts of total TGF-β. Recombinant human TGF-β1 (PreproTech, Hamburg, Germany) was used as standard. The resulting SEAP (secreted alkaline phosphatase) activity in the culture supernatant was measured by adding an equal volume of pNPP buffer (0.2% 4-nitrophenyl-phosphate, 50 mM glycine, 1 mM MgCl_2_, 100 mM TRIS, pH 10.5) at 37°C. Resulting formation of 4-nitrophenol (pNP—yellow color) was determined photometrically at λ = 405 nm.

### Statistical analysis

All experiments were performed in duplicate using at least four different donors. Values were expressed as either mean ± SD or mean ± SEM. Statistical comparisons were performed by Student’s t test. A statistical difference was considered significant if *p* < 0.05.

## Results

### Effect of suspension and adhesion culture on the proliferative activity of PCMO

**Fig 1:**
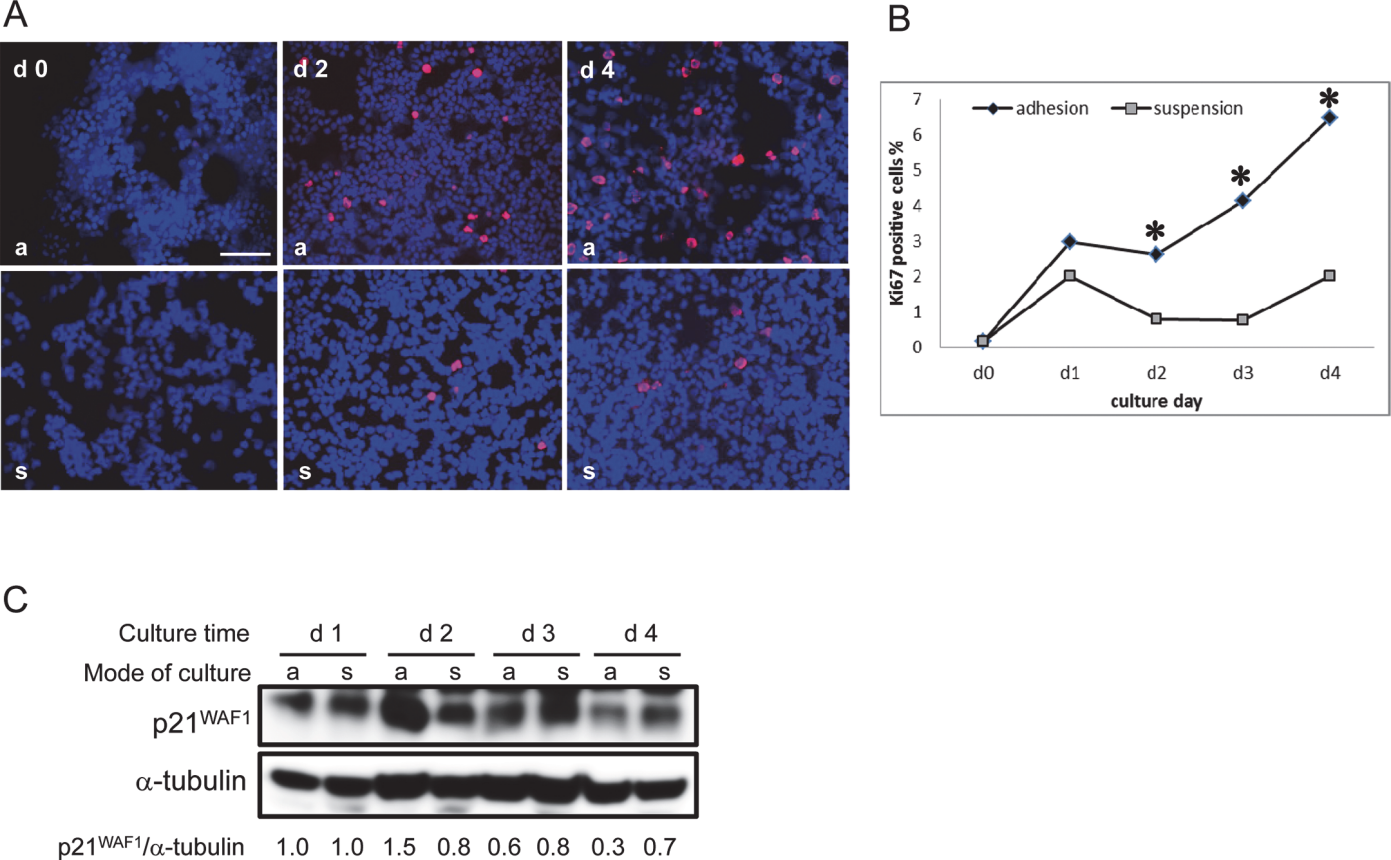
PCMO resume proliferation during a 4-day culture period. Peripheral blood monocytes cultured in 6-well plates were cultured for up to 4 days (d) under adherent (a) or suspended (s) growth conditions and subjected on the indicated days to staining for Ki67 and immunoblotting of p21^WAF1^. *Bar*, 50 μm. (A) *In situ* staining of PCMO cultures with Ki67 (red) and DAPI (blue). (B) Quantification of Ki67/DAPI-double positive cells in adhesion and suspension cultures from four different donors. Standard deviations were below 15%. *, p<0.05. (C) kinetics of p21^WAF1^ expression. The bands for p21^WAF1^ and α-tubulin, used as a loading control, were densitometrically analyzed. Displayed are the normalized values for p21^WAF1^ relative to those on day 1 set arbitrarily at 1.0, from one representative donor out of four different donors analyzed in total.

Principally, monocytes can be maintained in suspension or can be allowed to adhere to solid surfaces. In order to optimize culture conditions for accelerated growth, we analyzed in cultures of adherently and non-adherently growing monocytes/PCMO (prepared by density gradient centrifugation) the number of mitotically active cells. For this purpose, monocytes/PCMO growing adherently on tissue culture plastic or those from parallel cultures growing in suspension (adhesion prevented by an underlayer of agarose) were stained *in situ* with the proliferation marker Ki67 (Fig. 1A). Interestingly, we found that the fraction of Ki67-positive monocytes was higher under adherent conditions with significant differences on days 2, 3, and 4 (Fig. 1B). That this is indicative of mitotically active cells is also evident from a down-regulation (beyond day 2 of culture) of the cyclin-dependent kinase inhibitor p21^WAF1^. This protein mediates the growth-inhibitory effects of TGF-β1 on monocytes [21–23]. The p21^WAF1^ levels dropped below those in suspended cells cultured in parallel for the same length of time (Fig. 1C). The data suggest that adherent PCMO are more proliferation-active than their counterparts growing in suspension.

### Expression of activin and TGF-β receptors in PCMO

As outlined above, activins and TGF-βs are potential candidates in regulating pluripotency marker expression and self-renewal/proliferation in PCMO culture. To evaluate the possibility that PCMO can respond to activin(s) and/or TGF-β(s) in an autocrine fashion, we confirmed expression of type I and II receptors for activins (ALK4, ALK7, ActRIIA) and TGF-βs (ALK5, TβRII) during adherent PCMO culture by RT-PCR (Table 1). The quantitative RT-PCR (qPCR) data show that the five receptors were expressed at all time points analyzed. Interestingly, while expression of ALK7, ALK5, and TβRII decreased over culture time (starting on days 1, 0, and 2, respectively), expression of both ALK4 and ActRIIA strongly increased to reach peak levels on days 3–4 of culture (Table 1). These results show that PCMO are capable of responding to activins and TGF-β and suggest that the sensitivity of PCMO to activin(s) is enhanced on days 3–4 due to higher receptor expression.

**Table 1:** QPCR analysis of activin and TGF-β receptors during adherent PCMO culture.

	ALK4	ALK7	ActRIIA	ALK5	TβRII
day 0	1.00±0.00	1.00±0.00	1.00±0.00	1.00±0.00	1.00±0.00
day 1	3.18±0.36	2.26±1.50	3.59±2.14	0.27±0.18	1.51±0.21
day 2	3.11±1.32	0.95±0.38	2.35±1.04	0.70±0.49	1.53±0.47
day 3	4.67±2.64	0.54±0.13	4.71±2.78	0.95±0.54	1.07±0.47
day 4	4.41±0.63	0.26±0.05	2.19±1.17	1.31±0.44	0.58±0.03
day 6	2.33±0.86	n.d	n.d.	n.d.	n.d.

Values represent the normalized mean ± SEM (n = 4) and are given as fold-change in comparison with the respective gene expression levels in monocytes cultured for 1 h (day 0); n.d., not determined.

### Activin A and TGF-β1 synthesis and secretion during conversion of monocytes to PCMO

**Fig 2:**
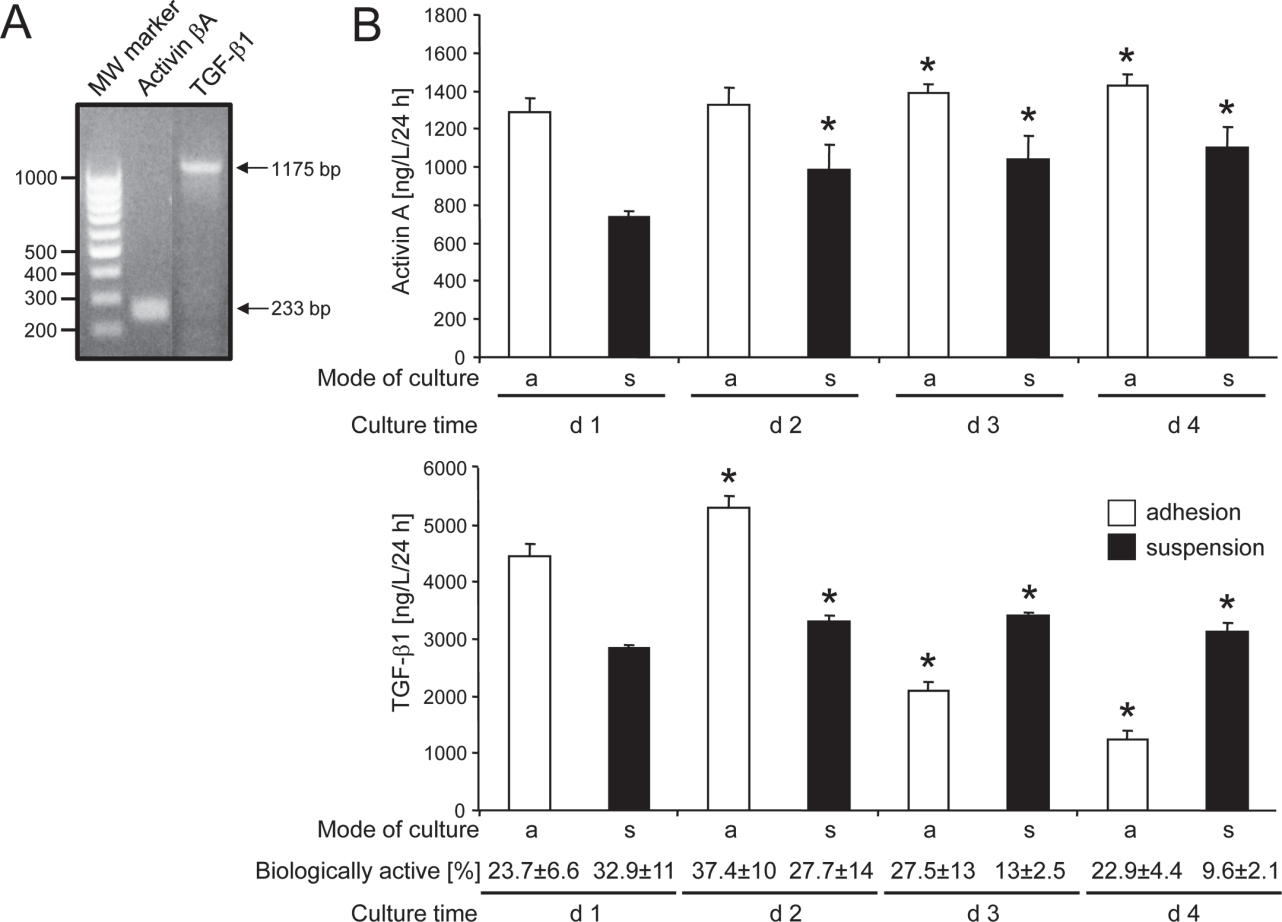
Activin A and TGF-β1 synthesis and secretion during conversion of monocytes to PCMO. (A) Standard endpoint RT-PCR-based detection for the β_A_-subunit of activin and TGF-β1 in PCMO. MW marker, molecular weight marker = 100 bp ladder. (B) Activin A and TGF-β1 levels in culture supernatants of monocytes/PCMO growing adherently (a) or in supsension (s) at different time points during culture. Supernatants, conditioned for 24 h, were taken every day until day 4 and subjected to ELISA specific for activin A or total TGF-β1, and bioassay for TGF-β. Data were calculated after subtraction of background activin A and TGF-β levels (contained in the AB serum) and represent means ± SD from five different donors normalized to 10,000 cells. Asterisks indicate a significant difference relative to the respective levels on day 1. Numbers below the graph indicate the percentage of active TGF-β in the respective samples as determined by bioassay (means ± SD). Differences were significant between day 2 and day 4 in both adherent and suspended cells.

Next we monitored, this time by standard endpoint RT-PCR, PCMO for expression of the β_A_ subunit of activin (contained in activin A and AB) and TGF-β1 (Fig. 2A). Our data show that mRNA for both proteins is present in day 4 PCMO. To determine whether monocytes/PCMO also secrete activin A and TGF-β1 into the culture medium, we measured by specific ELISAs the concentrations of both growth factors in the culture supernatants of PCMO growing either adherently or in suspension (Fig. 2B). Levels of activin A remained unchanged or even increased in supernatants from adhesion and suspension cultures, respectively, though levels in supernatants from adherent cells were higher at all time points analyzed (Fig. 2B, upper graph). Like activin A, total TGF-β1 protein was detectable at all time points and regardless of growth conditions. However, in the cultures of adherent cells, levels were initially high, peaked on day 2 but subsequently sharply declined on day 4 to only 23.6% of the level on day 2 (Fig. 2B, lower graph). In suspended cells, total TGF-β1 levels were lower on day 1 and marginally increased up to day 4 (Fig. 2B, lower graph). Using a sensitive bioassay, we determined the fraction of bioactive TGF-β to range between 9.6 and 37.4% of the total amount of TGF-β. Interestingly, the percentage of active TGF-β (or the ratio of active:total TGF-β declined over culture time, and in both adherent and suspended cells was significantly lower on day 4 when compared to day 2 (Fig. 2B, lower graph). TGF-β2 and TGF-β3 were not detectable (data not shown). We conclude that PCMO are capable of producing and secreting activin A and active and latent forms of TGF-β1 into the culture medium.

### PCMO respond to exogenous activins and TGF-β1 with activation of Smad2

**Fig 3:**
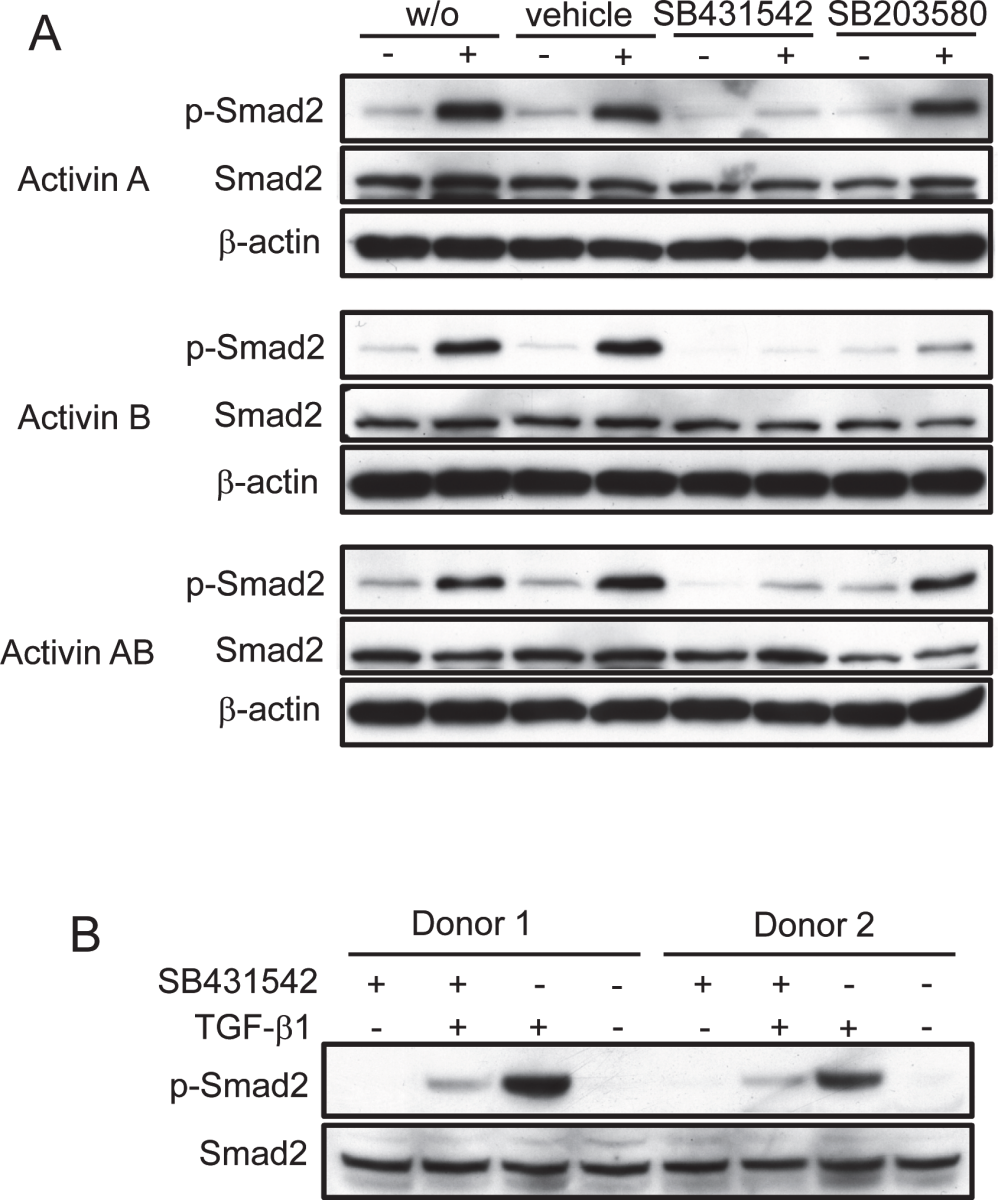
PCMO respond to exogenous activins and TGF-β1 with phosphorylation of Smad2. (A) PCMO were stimulated on day 4 of culture with 50 ng/ml of either activin A, activin B, or activin AB in the presence or absence of the ALK4/5/7 inhibitor SB431542 (1 μM), the p38 MAPK inhibitor SB203580 (10 μM), vehicle (dimethylsulfoxide, 0.1%), or medium alone (w/o) for 1 h followed by immunoblotting for phosphorylated Smad2C (p-Smad2), total Smad2 (Smad2), and β-actin as a loading control. (B) Responsiveness of PCMO from two different donors to TGF-β1 stimulation. PCMO were stimulated on day 4 with 5 ng/ml TGF-β1 in the presence or absence of SB431542 (1 μM), or vehicle for 1 h followed by immunoblotting for p-Smad2C and Smad2. Data in A and B are representative of four different donors.

Activin A controls stem cell function [9–11], while activins B and AB promote differentiation of mESCs in insulin-producing cells [27, 28] and bind to ActRIIA and ALK7 to mediate insulin secretion from pancreatic β-cells [29]. Since we routinely generate insulin-producing cells from PCMO, studying activin B and AB signaling is of great interest. To test whether PCMO respond to activins and TGF-β with intracellular signaling, we stimulated PCMO on day 4 with recombinant activins A, B, or AB (Fig. 3A), or TGF-β1 (Fig. 3B). Notably, PCMO reacted to all three activin isoforms with phosphorylation of Smad2C (Fig. 3A), while phosphorylation of Smad3C was not detectable (S2 Fig.). Notably, even unstimulated control cells contain readily detectable levels of phospho-Smad2C (Fig. 3A). In monocytes stimulated with activin A, we also noted phosphorylation of p38 MAPK (data not shown) which was inhibited by the p38 MAPK inhibitor SB203580 but not by the structurally related SB431542, a small molecule inhibitor with high selectivity for the type I receptors ALK5, ALK4, and ALK7 [30, 31] (data not shown). Conversely, SB431542 but not the SB203580 blocked Smad2 activation induced by either activin A, activin B, or activin AB (Fig. 3A). As shown earlier by us [Ref. 32: Fig. 3B], PCMO, but not monocytes, also responded to exogenous TGF-β1 with phosphorylation of Smad2C. Here, we confirm these findings and show, in addition, that TGF-β1-induced Smad2C phosphorylation is inhibited by SB431542 (Fig. 3B). However, as demonstrated in a previous publication [Ref. 32: Fig. 3B] TGF-β1-induced phospho-Smad3 was almost undetectable in day 6 PCMO. These data show that PCMO are capable of responding to exogenous (and presumably also endogenous) activins and TGF-β1 with activation of Smad2C but not Smad3C (presumably as a result of low Smad3 expression already on day 4, see S2 Fig.), and that this activation is mediated by ALK4 and ALK5, respectively. Moreover, activin-induced Smad2 phosphorylation is independent of the activation of p38 MAPK.

### Kinetics of endogenous Smad activation in PCMO culture and its relation to endogenous activin A and TGF-β1 secretion

**Fig 4:**
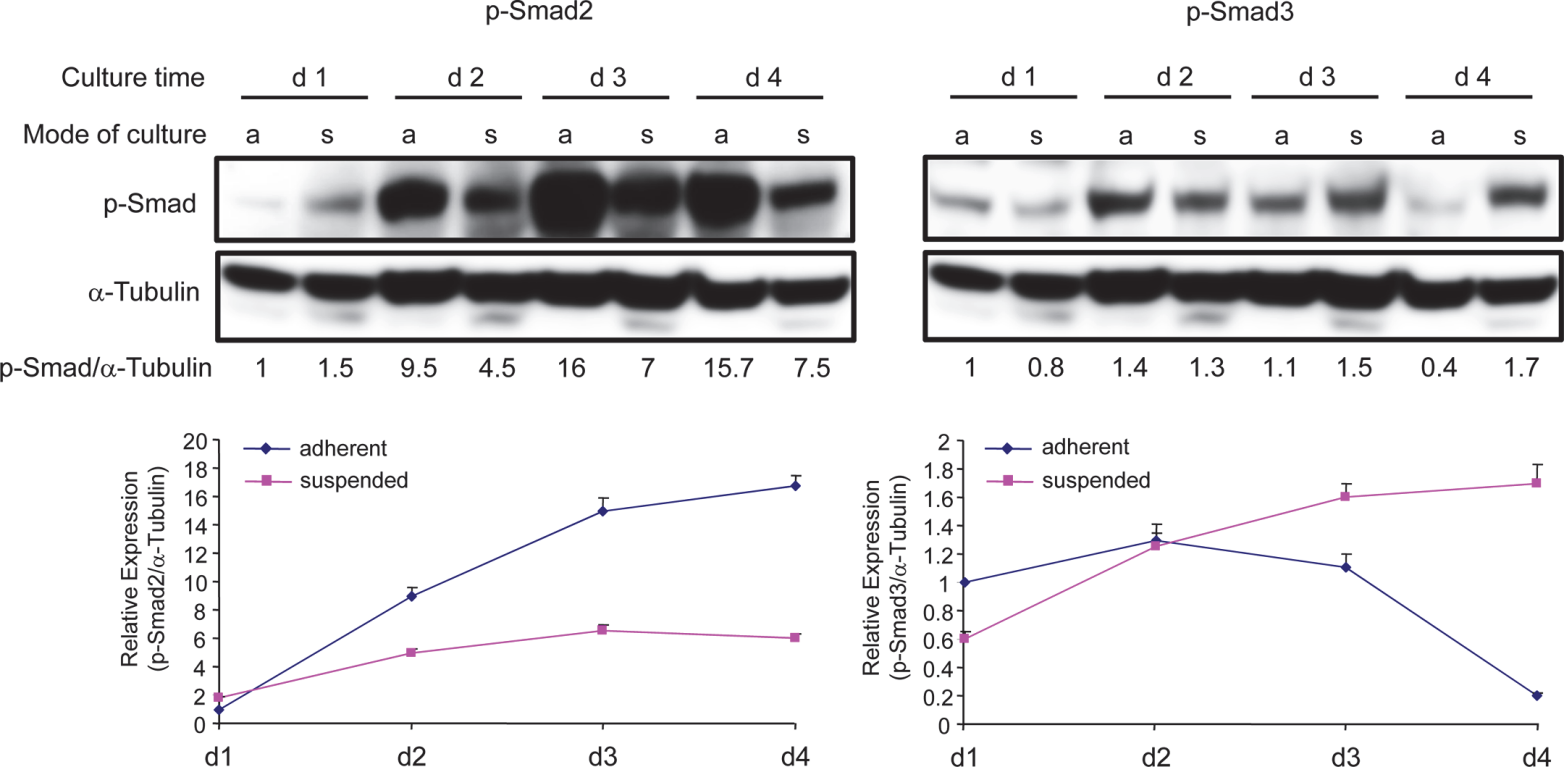
Kinetics of Smad activation during PCMO culture. The activation state of Smad2 (left) and Smad3 (right) in adherent (a) and suspended (s) monocytes/PCMO was assessed at various time points by immunoblotting and ELISA (graphs below blots) for p-Smad2(Ser465/467) and p-Smad3(Ser423/425). Alpha-tubulin was used for normalization of p-Smad2 and p-Smad3 signal intensities from blots and for the respective ELISA data (means ± SD). The p-Smad3 blots had to be exposed for a much longer time than the p-Smad2 blots due to the low expression of Smad3. Numbers below the blots indicate the densitometric values for phospho-Smads normalized to those for α-tubulin. Data are representative of four different donors.

Previous data from our group have shown that during conversion to PCMO the monocytes’ response towards exogenous TGF-β1 is changing with respect to Smad2 and Smad3 activation in such a way that they become sensitive to Smad2C and insensitive to Smad3C phosphorylation [32]. Given the presence of activin A and TGF-β1 in the culture supernatants and thus the existence of a self-stimulatory/autocrine signaling loop in these cells, we hypothesised that the sensitivity of PCMO to endogenous Smad-activating agents is altered in a similar way. In accordance with this assumption, we observed a strong and selective upregulation of phospho-Smad2C in adherent cells until day 4 as measured by both phosphoimmunoblotting and ELISA (Fig. 4, left panel). This increase in phospho-Smad2C may have been stimulated by endogenous activin secretion in combination with upregulation of activin receptor expression rather than TGF-β(s), the levels of which declined between day 2 and 4 (see above).

Above we have shown that activin A levels in supernatants from adherent cells were higher than those from suspension cells. If endogenous activin were responsible for the generation of phospho-Smad2C, then the amounts of phospho-Smad2C should be lower in suspended cells. In agreement with this prediction, we observed that phospho-Smad2C levels in suspended cells were much weaker than in adherent cells (Fig. 4, left panel). This argues in favor of endogenously produced activin as the Smad2 activation-inducing agent (see Discussion).

The upregulation of phospho-Smad2C was paralleled by a decrease in the levels of phospho-Smad3C in adherent but not in suspended cells (Fig. 4, right panel). This was paralleled, as shown above, by a decline in the levels of both secreted total TGF-β1 and p21^WAF1^ expression between culture day 2 and 4 in adherent but not in suspended cells. Since activins were unable to activate Smad3 (see S2 Fig.), this argues in favor of endogenously produced TGF-β(s) as the Smad3 activation-inducing agent (see Discussion).

### Identification of autocrine ligands that upregulate pluripotency marker expression and suppress PCMO proliferation

**Fig 5:**
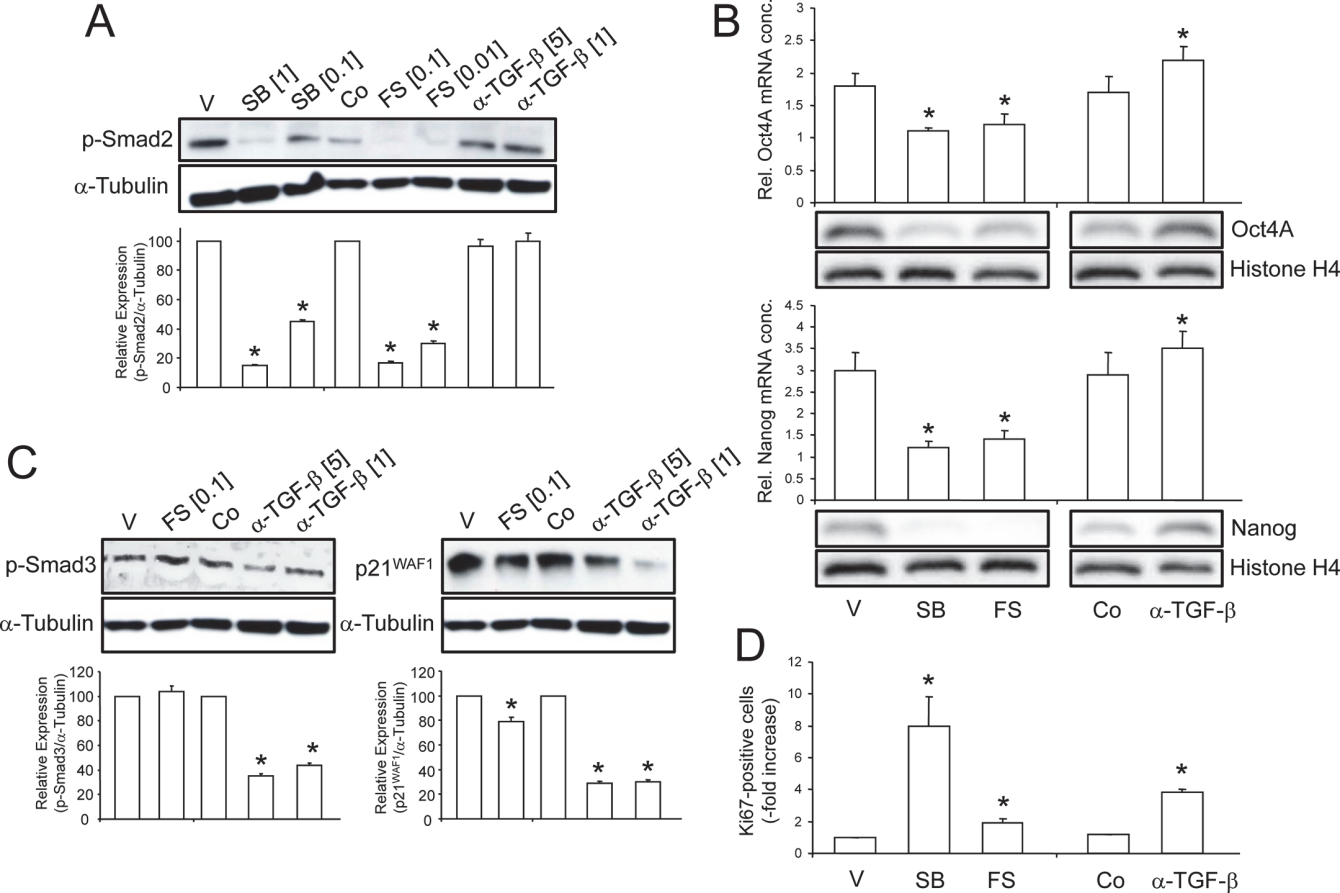
Identification of the autocrine ligands that regulate pluripotency gene expression and proliferation in PCMO. Effect of SB431542 (SB), follistatin (FS), and anti-TGF-β antibody (α-TGF-β) on day 4 of culture on (A) Smad2C phosphorylation as determined by immunoblotting and ELISA (graph below blot). Co, isotype control. Numbers in brackets indicate the concentrations used (μM for SB and μg/ml for FS and α-TGF-β. (B) Oct4A and Nanog expression as determined by qPCR (graphs) and immunoblotting. Oct4 and Nanog were immunodetected in nuclear proteins (25 μg/lane) from the same day 3 (Oct4) and day 4 (Nanog) PCMO used for qPCR analysis. Successful enrichment of nuclear proteins and equal protein loading was verified with an antibody to histone H4. (C) Effect of FS, and α-TGF-β on Smad3C phosphorylation and p21^WAF1^ expression in day 4 PCMO as determined by immunoblotting and ELISA (graphs below blots). (D) Ki67 expression on day 4 of culture. In (B) and (D), SB, FS, and α-TGF-β were used at concentrations of 1 μM, 0.1 μg/ml, and 5 μg/ml, respectively. ELISA data in A and C, and qPCR data in B and D are means ± SD from triplicate samples and were derived from one donor. Data are representative of four different donors. Asterisks indicate a significant difference between vehicle and SB or FS-treated cells and between Co and α-TGF-β-treated cells.

Notably, the mRNA expression kinetics of the activin target genes Nanog and Oct4A [Ref. 4: Fig. 5] paralleled that of Smad2 activation in adherent cells (see Fig. 4), suggesting the possibility that endogenous activin(s) and/or TGF-β(s) sustain phospho-Smad2C levels and pluripotency marker expression in PCMO. To prove that functionally, we blocked endogenous TGF-β/activin signaling at both the receptor and the ligand level. Specific neutralization of activin’s and TGF-β‘s biological activity with follistatin and anti-TGF-β1,-β2,-β3 antibody (anti-TGF-β antibody), respectively, and the ALK4/5 inhibitor SB431542, was performed in order to interfere with a possible autocrine stimulation of these growth factors. Monocytes cultured in PCMO medium were treated for 4 days with either vehicle (dimethylsulfoxide), SB431542, follistatin, isotype control, or anti-TGF-β antibody and analyzed for Smad2 activation by immunoblotting and ELISA. Both SB431542 and follistatin but not anti-TGF-β antibody effectively inhibited Smad2C phosphorylation (Fig. 5A). Next, we assessed the effects of various inhibitors of activin/TGF-β signaling on Oct4A on day 3 and Nanog expression on day 4 using both qPCR and immunoblotting. Interestingly, cells treated with SB431542, or follistatin, but not cells treated with vehicle, isotype control, or anti-TGF-β antibody, failed to upregulate Oct4A and Nanog (Fig. 5B). The suppressing effect of SB431542 was stronger than that of follistatin (Fig. 5B). Depletion of TGF-βs even had a small but statistically significant stimulatory effect on Oct4A and Nanog expression (Fig. 5B).

To evaluate the possibility that endogenous TGF-βs sustain phospho-Smad3C levels and growth inhibition in PCMO, we blocked endogenous TGF-β signaling at the ligand level using anti-TGF-β antibody. Monocytes cultured in PCMO medium were treated for 4 days with either follistatin, isotype control, or anti-TGF-β antibody and analyzed by immunoblotting and ELISA for Smad3 activation and p21^WAF1^ expression. Anti-TGF-β antibody was able to inhibit Smad3C phosphorylation which was particularly apparent for the higher concentration (5 μg/ml) (Fig. 5C). Likewise, anti-TGF-β antibody treatment resulted in lower expression of p21^WAF1^, again revealing a strong correlation with TGF-β levels (p21^WAF1^ was down-regulated during adherent PMO culture (see Fig. 1C) concomitant with TGF-β levels in the medium (see Fig. 2B)). Interestingly, treatment with 100 ng/ml follistatin also weakly reduced p21^WAF1^ levels (Fig. 5C).

We then assessed the effects of SB431542 and anti-TGF-β antibody on growth, postulating that both should relieve the cells from growth inhibition. Interestingly, in cells treated with SB431542 the numbers of Ki67-positive cells increased relative to vehicle treated control cultures on day 4 by ~8-fold (Fig. 5D). Notably, anti-TGF-β antibody, but not the isotype control, resulted in a 3.8-fold increase in the number of Ki67-positive cells (Fig. 5D). Consistent with its ability to moderately suppress p21^WAF1^ levels (Fig. 5C), follistatin also increased the number of Ki67-positive cells albeit only by ~2-fold (Fig. 5C), indicating that activin A is also growth-inhibitory in monocytes/PCMO.

We conclude from these data that activins promote, while TGF-β(s) inhibit Oct4A and Nanog expression. With respect to growth regulation, ALK4 and/or ALK5 inhibition, or anti-TGF-β antibody or follistatin treatment removes from the cells a growth constraint induced primarily by autocrine TGF-βs and, to a lesser extent, by autocrine activins via p21^WAF1^.

### Analysis of cell cycle regulating genes in PCMO following inhibition of endogenous activin(s) and TGF-β(s)

**Fig 6:**
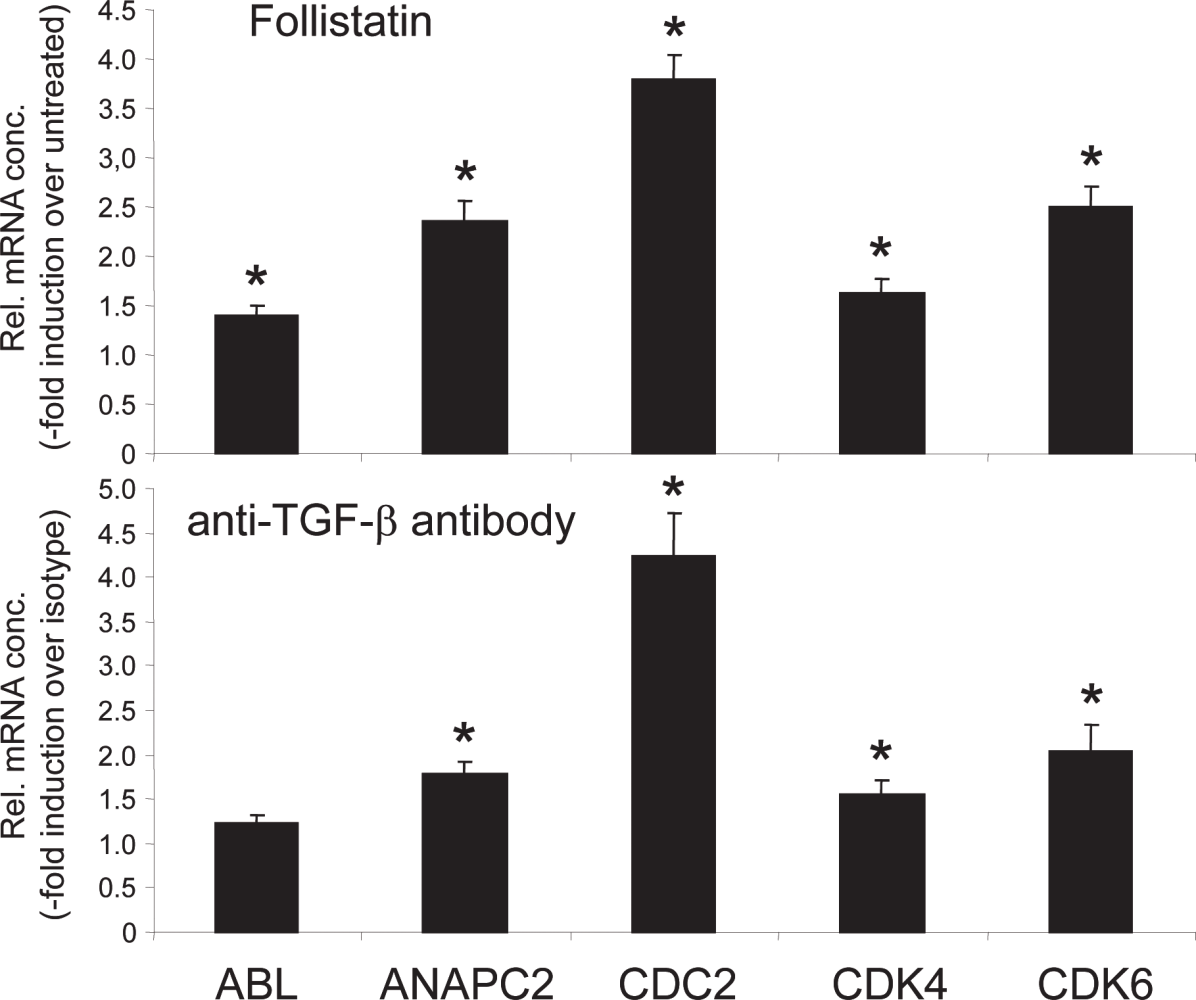
Expression of cell cycle regulating genes in PCMO following neutralization of endogenous activin(s) and TGF-β(s). QPCR-based detection of the indicated genes after treatment of PCMO cultures with follistatin or anti-TGF-β antibody. Data represent the-fold stimulation relative to untreated cells (follistatin) or isotype IgG1-treated control cells (anti-TGF-β antibody) on day 4 of culture, mean ± SD. Data (means ± SD from triplicate samples) are from one donor and are representative of four different donors. ANAPC2, anaphase promoting complex subunit 2; CDC2, cell division control protein 2; CDK, cyclin-dependent kinase.

In order to understand the effect of neutralizing TGF-βs) or activin(s) on mitotic activity at the molecular level, we analyzed by qPCR regulation of various cell cycle regulatory proteins at day 4 of culture (Fig. 6). We found upregulation of c-ABL tyrosine kinase by follistatin (1.4-fold relative to isotype IgG1-treated controls) and by anti-TGF-β antibody (1.22-fold), anaphase promoting complex subunit 2 (follistatin: 2.36-fold, anti-TGF-β antibody: 1.79-fold, cell division control protein 2 (follistatin: 3.8-fold, anti-TGF-β antibody: 4.23), cyclin-dependent kinase 4 (follistatin: 1.63, anti-TGF-β antibody: 1.55), cyclin-dependent kinase 6 (follistatin: 2.5-fold, anti-TGF-β antibody: 2.03-fold). These data suggest enhanced mitotic activity of PCMO following inhibition of TGF-β(s) and activin(s) in the culture medium and confirm our conclusion that both autocrine TGF-β(s) and activin(s) play a crucial role in suppressing PCMO mitotic activity in standard PCMO culture.

## Discussion

In a series of studies we have shown that a subset of peripheral blood monocytes can be coaxed *in vitro* to become a stem cell-like cell type termed PCMO. These cells re-express, amongst other stem cell markers, Oct4A and Nanog, the expression of which peaked at days 3–4 [4]. Moreover, these cells resumed mitotic activity when cultured under adherent conditions as measured by cell counting, thymidine incorporation [3], activation of the proliferation-associated MAPK ERK1/2 [6] as well as Ki67 staining (this study). This may suggest the possibility that Oct4A/Nanog expression and mitotic activity are controlled by the same or related factors.

Stemness and self-renewal of human ES cells are controlled by members of the TGF-β/activin/BMP family of growth and differentiation factors, particularly by activin/Nodal [8–12]. Unlike its role in murine ES cells, TGF-β is known to act on monocytes/macrophages as a suppressor of growth, an effect which is mediated primarily through p21^WAF1^ and Smad3 [23]. Given the stem cell-like features of PCMO, we hypothesised that particularly with respect to activin signaling similar regulatory loops as in ES cells might operate in PCMO. An earlier PCR-based screening in PCMO for expression of members of the TGF-β superfamily of ligands which signal through ALK4 or ALK5, namely Nodal, Gdf3, Lefty A, and Lefty B, was negative [4], lending support to the notion that endogenous activin and TGF-β signaling is responsible for regulation of pluripotency and growth in standard PCMO cultures. Interestingly, autocrine activin signaling appears to be induced during PCMO culture as evidenced by an increase in i) the concentration of activin A in the culture supernatant, ii) the expression of ALK4 and ActRIIA by PCMO, iii) the activated (phosphorylated) form of Smad2 in PCMO, and iv) the responsivity of monocytes to exogenous activins as assessed by Smad2 (and less prominently Smad3) activation. Notably, the strong and selective upregulation of phospho-Smad2C in adherent cells correlated well with higher levels of activin A and increased activin receptor expression. Additional evidence for Smad2 activation resulting from enhanced activin receptor stimulation also came from the observation that in suspended cultures both secreted activin A and cellular phospho-Smad2C levels were lower and did not increase during culture. Due to its rapid decline in the culture supernatants between day 1 and 4, TGF-β is unlikely to contribute to the increase in phospho-Smad2C levels. The assumption that Smad2 activation is induced by activin rather than TGF-β was confirmed by the finding that only follistatin and SB431542, but not anti-TGF-β1/2/3 antibody, effectively suppressed Smad2C phosphorylation.

Follistatin and SB431542 but not anti-TGF-β antibody also inhibited upregulation of Oct4A and Nanog, providing experimental evidence pluripotency is mediated by endogenous activin/Smad2 signaling. Since only expression of ALK4 (which preferably binds to activin A), but not ALK7 (which preferably binds to activin AB and B), was strongly induced during culture, activin A may be the relevant activin isoform in PCMO cultures, although PCMO are responsive to all three activin isoforms (see Fig. 4).

TGF-β1 levels in the cultures negatively correlated with Oct4A and Nanog expression (TGF-β1 declined from day 2 to day 4, while Oct4A/Nanog rose and peaked at days 4–5 [4] and antibody-mediated neutralization of TGF-βs in the cultures, in contrast to follistatin, slightly increased both Oct4A and Nanog expression (see Fig. 5B). This suggests that TGF-β signaling is inhibiting rather than promoting pluripotency. Along the same lines are findings that TGF-β signaling antagonized reprogramming efficiency during generation of iPS cells from fully differentiated somatic cells, since blockage of TGF-β signaling by antibody-mediated depletion of ligands or SB431542-mediated inhibition of ALK4/5/7, significantly increased the reprogramming efficiency [19, 20]. This is noteworthy, since as outlined above, PCMO generation appears to resemble the process of reprogramming of somatic cells to iPS cells. Interestingly, p21^WAF1^ not only arrests monocytes in G1/S in response to TGF-β, but also has a role in differentiation of human peripheral blood monocyte precursors to macrophages and functional dendritic cells [33], and to osteoclasts [34, 35]. Hence, its downregulation is not only compatible with relief from growth inhibition but may also confer enhanced pluripotency. Another issue related to this is as to whether the decline in autocrine TGF-β levels, either on its own or because it allows for enhanced activin signaling, is causal for an enhanced reprogramming efficiency of PCMO.

**Fig 7:**
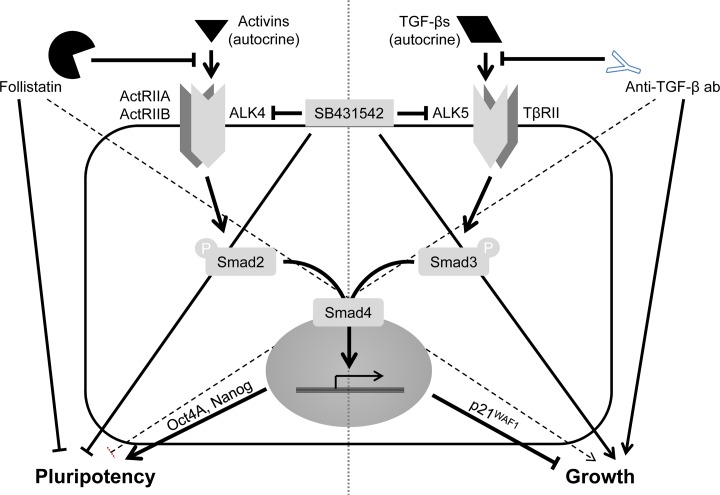
Cartoon to illustrate the effects of activins and TGF-βs, their receptors and target genes, and their inhibitors, on pluripotency and growth of adherently growing PCMO. Stimulatory (↓) and inhibitory (⊥) interactions are depicted by bold and dashed lines to indicate strong and weak interactions, respectively. Ab, antibody.

We have also characterized the underlying growth regulatory circuit in the PCMO cultures. TGF-β is known for its growth-inhibitory function on monocytes [21–23]. Moreover, during an earlier study comparing the effect of different sera on PCMO growth, we observed that the TGF-β1 levels in culture supernatants correlated negatively with PCMO yield [7]. This led us to speculate that autocrine production of TGF-β plays a role in growth regulation during early PCMO culture. However, in contrast to activin A, the levels of TGF-β1 in the culture supernatants were initially high but rapidly declined between days 2 and 4 of culture (see Fig. 2B), with a parallel decrease in phospho-Smad3C and p21^WAF1^ protein levels in these cells. A loss of the phospho-Smad3C response to exogenously applied TGF-β1 (5 ng/ml for 1 h) during the monocyte → PCMO conversion was observed by us earlier [Ref. 32: Fig. 3B]. The phosphorylated Smad3C was unlikely to be induced by endogenously produced activin(s) since stimulation of day 4 PCMO with recombinant activin A, B, or AB failed to activate Smad3C (see S2 Fig.). Final proof that both phosphorylation of Smad3C and expression of p21^WAF1^ protein were induced by autocrine TGF-βs rather than activins came from the ability of the anti-TGF-β antibody to effectively suppress both responses. As a consequence of reduced TGF-β/Smad3 signaling, cells experience a relief from growth inhibition as evidenced by an increase in the number of Ki67-positive cells and the induction of various cell cycle-regulatory genes. Interestingly, follistatin treatment also induced a ~2-fold increase in the number of Ki67-positive cells. This smaller increase compared to treatment with anti-TGF-β antibody (~3.8-fold) correlated with an only moderate decrease in p21^WAF1^ levels. From the observation that SB431542 induced a larger increase (~8-fold) in Ki67-positive cells compared to treatment with either follistatin or anti-TGF-β antibody alone, we conclude that the observed promitotic effect of SB431542 was due to inhibition of both TGF-β *and* activin signaling. The lack of an additive effect can easily be explained with the supposedly more complete pharmacologic inhibition by SB431542 compared to neutralization of TGF-βs and activins in the culture supernatant. The various proteins, their inhibitors, and the proposed regulatory interactions between them are summarized in Fig. 7.

In a separate project, we are currently attempting to elucidate the roles of M-CSF and IL-3 in the expression of activin and TGF-β and their receptors. Preliminary evidence suggests that M-CSF is not involved in TGF-β1 expression/secretion as we were unable to detect changes in TGF-β1 levels following omission of M-CSF or addition of M-CSF to concentrations of up to 100 ng/ml (H.U., unpublished observation).

## Conclusions

Our data suggest that the decrease in phospho-Smad3C is the result of reduced TGF-β signaling due to reduced TGF-β secretion, while the increase in phospho-Smad2C is likely to reflect increased activin signaling due to higher activin secretion and/or the increasing activin receptor expression in the cultures. This suggests that Oct4A/Nanog expression is primarily mediated by enhanced activin/Smad2 signaling while *de novo* proliferation is the result of decreased TGF-β/Smad3 signaling.

## Supporting Information

S1 Fig Morphology of PCMO and NeoHeps, and expression of hepatocyte markers by Neoheps. (TIF)

S2 Fig PCMO fail to respond to exogenous activins with phosphorylation of Smad3C. (TIF)
