# Supplementary figures and images for: Pluripotency Gene Expression and Growth Control in Cultures of Peripheral Blood Monocytes during Their Conversion into Programmable Cells of Monocytic Origin (PCMO): Evidence for a Regulatory Role of Autocrine Activin and TGF-β
Source: PLoS One. 2015 Feb 23;10(2):e0118097. doi: 10.1371/journal.pone.0118097 (PMC4338298; doi:10.1371/journal.pone.0118097)

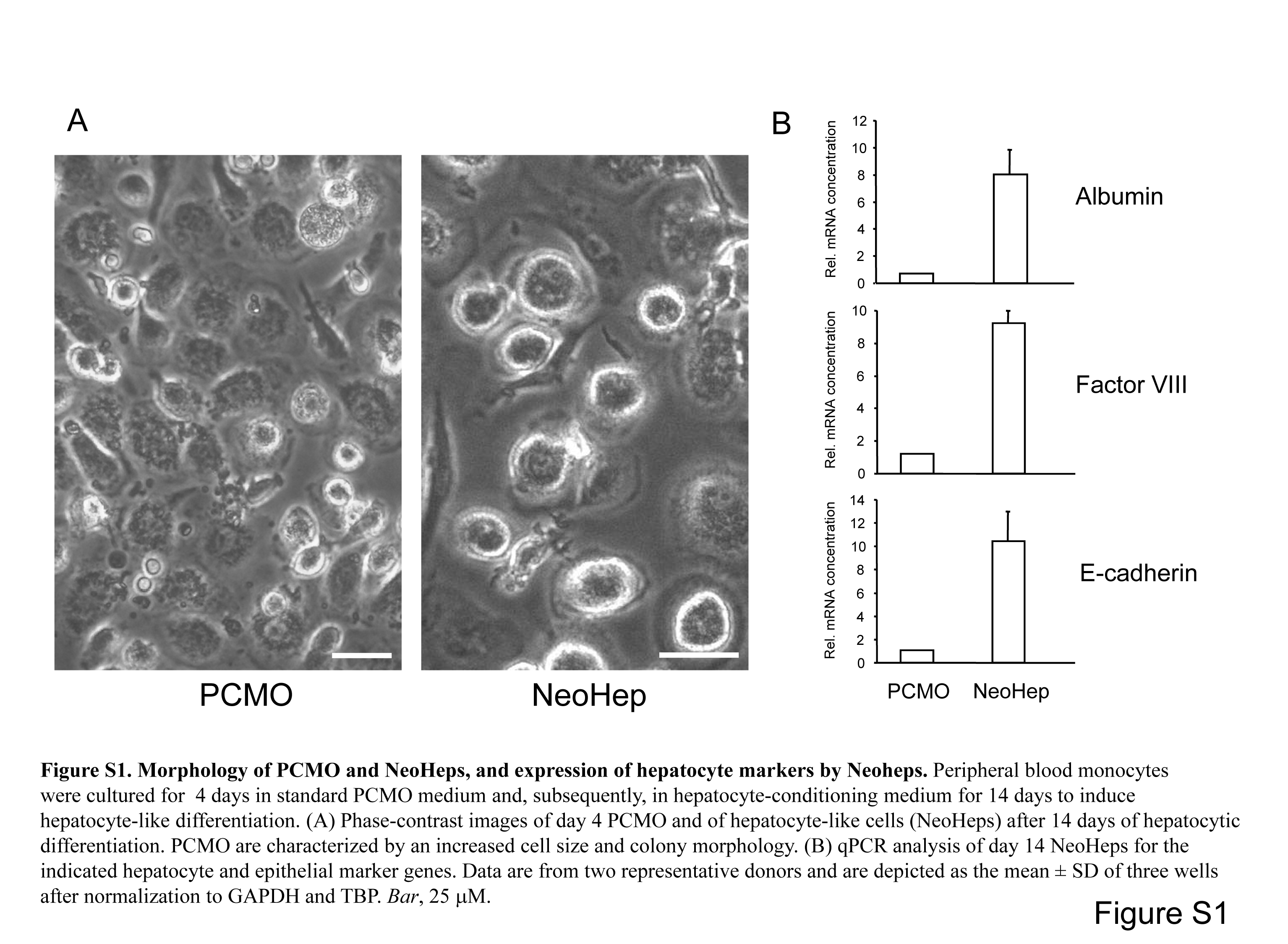

Supplement: S1 Fig — (TIF) [file pone.0118097.s001.tif]

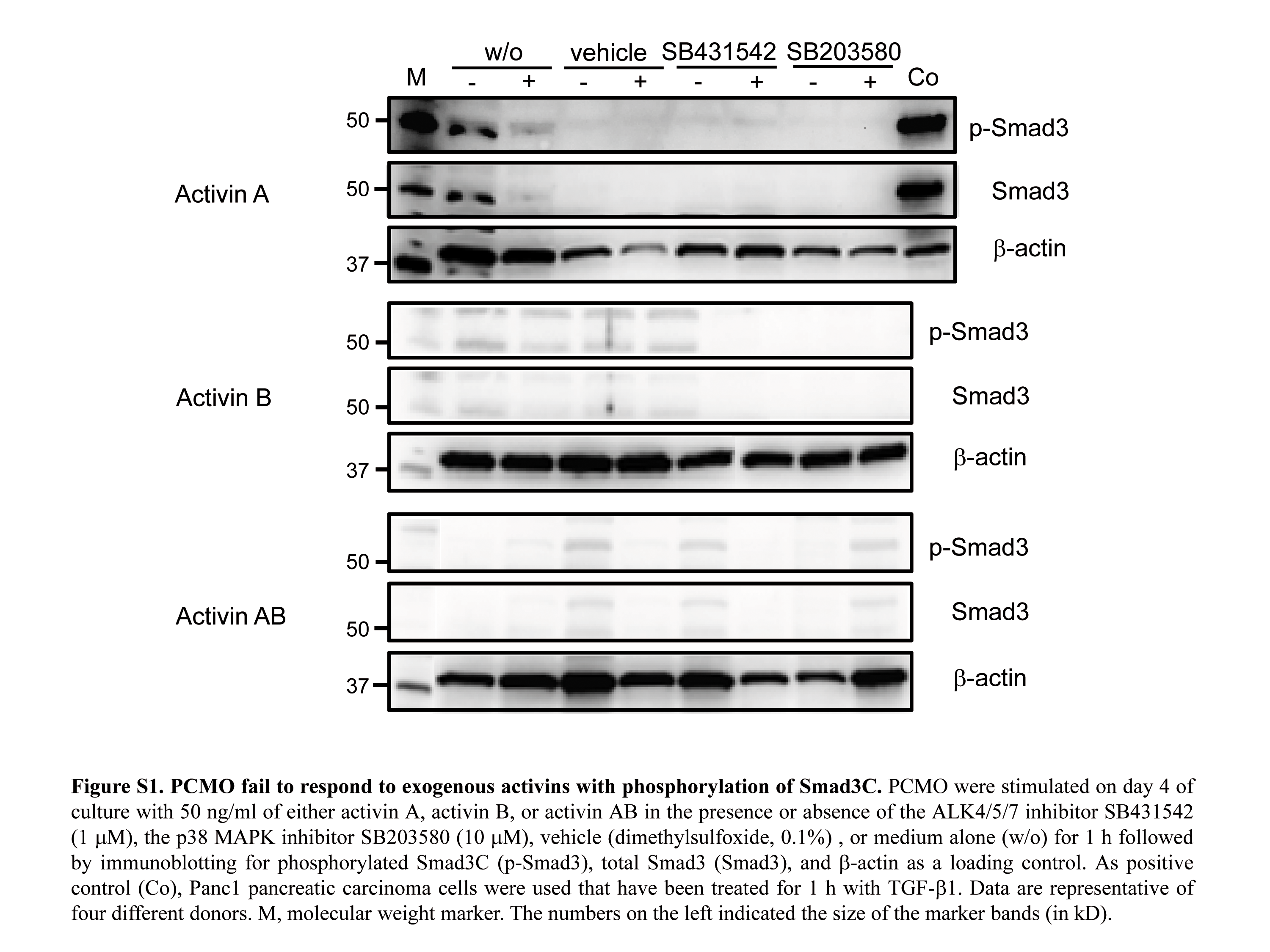

Supplement: S2 Fig — (TIF) [file pone.0118097.s002.tif]
